# Sublethal Concentration of Chloramphenicol Threatens the Health of *Bombus terrestris* by Regulating Gene Expression, Altering Enzyme Activity and Disrupting Gut Microbiota

**DOI:** 10.3390/ijms27136004

**Published:** 2026-07-04

**Authors:** Zhu Qin, Shuai Guo, Shuang Wang, Xi Xu, Haijun Bai, Bian Zhao, Cheng Liang, Kun Dong, Xueyang Gong, Yakai Tian

**Affiliations:** 1 Eastern Bee Research Institute, College of Animal Science and Technology, Yunnan Agricultural University, Kunming 650201, Chinadongkun19722004@aliyun.com (K.D.); 2 Sericulture and Apiculture Research Institute, Yunnan Academy of Agricultural Sciences, Mengzi 661100, China; 18559196339@163.com (B.Z.); ic@yaas.org.cn (C.L.)

**Keywords:** chloramphenicol, *B. terrestris*, sublethal effect, gut microbiota

## Abstract

Bumblebees are dominant pollinators threatened by environmental antibiotic residues. This study investigated sublethal chloramphenicol (12 and 120 μg/L) effects on *Bombus terrestris* after 15 days’ exposure. The results showed that chloramphenicol exposure had no significant effect on the survival rate and cumulative food intake of bumblebees, confirming the sublethal property of the tested concentrations. However, chloramphenicol significantly dysregulated the expression of genes related to learning–memory (*DopR2*, *Oamb*, *NMDA*), immunity (*abaecin*, *defensin*) and detoxification (*cyp9Q6*) in bumblebees. High-dose chloramphenicol significantly increased carboxylesterase activity and reduced malondialdehyde content, while superoxide dismutase activity remained unchanged. In addition, chloramphenicol exposure significantly reshaped the gut microbiota structure of bumblebees, reduced the abundance of core beneficial symbiotic bacteria, and increased the proportion of drug-resistant bacteria. Our findings indicate that sublethal concentrations of chloramphenicol can impair bumblebee health through multiple pathways, including regulating gene expression, altering antioxidant enzyme activity and disrupting gut microbiota homeostasis. This study provides multi-dimensional toxicological data and a scientific basis for the ecological risk assessment of agricultural antibiotic residues to pollinator insects.

## 1. Introduction

Pollinators are a core biological resource safeguarding global food security and terrestrial ecosystem stability, as they deliver critical pollination services for both agricultural crops and wild flowering plants. Pollinator declines can trigger the loss of these essential pollination services, which carry profound negative ecological and economic impacts that may significantly disrupt the maintenance of wild-plant diversity, wider ecosystem stability, crop production, food security and human welfare [1,2]. Among diverse pollinator taxa, bumblebees (*Bombus* spp.) serve as dominant pollinators for a wide range of cultivated crops (including Solanaceae and Leguminosae species) and wild flowering plants, owing to their unique adaptive traits such as low-temperature tolerance, extended foraging activity periods, and highly efficient buzz pollination. Their pollination efficiency is notably superior to that of honeybees and other pollinator groups, conferring bumblebees an irreplaceable ecological and economic role in both protected horticulture and natural terrestrial ecosystems [3]. As a widely distributed bumblebee species with strong environmental adaptability and a broad pollination host range, *B. terrestris* has been extensively commercialized and applied for greenhouse crop pollination worldwide, establishing it as an ideal model organism for exploring pollinator responses to various environmental stressors [4].

Widespread antibiotic residues in agricultural environments have become an emerging persistent pollutant worldwide, posing potential ecological risks to non-target organisms including pollinators [5]. Antibiotics are widely used in agricultural production not only for livestock breeding and disease prevention, but also for the control of bacterial and fungal diseases in crops, which greatly promotes agricultural yield increase but also leads to widespread residue accumulation in farmland ecosystems [6]. Chloramphenicol, a broad-spectrum antibiotic with high chemical stability and long environmental half-life, remains frequently detected in soil, water, nectar and pollen despite restricted use in food-producing animals in many regions [7,8]. During routine foraging activities, pollinators are exposed to environmental antibiotic residues via ingestion of contaminated pollen and nectar, as well as direct contact with contaminated soil and water sources. Critically, environmental residual concentrations of antibiotics predominantly exist at sublethal levels that do not elicit immediate insect mortality, but exert chronic, latent adverse effects on physiological metabolism, behavioral performance, reproductive capacity, and other vital biological processes [9]. Despite their concealed nature, such sublethal effects can indirectly undermine the survival adaptability and pollination efficiency of pollinators, thereby threatening the sustainability of agricultural production and the stability of natural ecosystems. Therefore, systematic investigations are urgently required to elucidate the potential ecological risks posed by sublethal antibiotic residues to pollinator health.

The physiological health and behavioral competence of bumblebees directly determine their pollination efficiency. Learning and memory capacity supports efficient foraging and stable pollination service quality [10], antioxidant and detoxification enzyme systems constitute the core defense against environmental pollutant stress [11], and gut symbiotic microbiota maintains host nutrient metabolism and immune homeostasis as the “second genome” [12]. As a broad-spectrum antibiotic once widely applied in livestock breeding and crop disease control, chloramphenicol features prolonged environmental residence time and high recalcitrance [13]. Even after its use in food-producing animals was restricted, it is still widely detected in soil, water, nectar and pollen, posing long-term potential ecological risks to pollinators. Mechanistically, chloramphenicol inhibits bacterial protein synthesis via the 50S ribosomal subunit and acts on both gram-positive and gram-negative bacteria, with distinct selective pressure on gut symbionts from tetracyclines [14]; studying its toxicity helps to comprehensively understand the disruptive effects of different antibiotics on pollinator gut microecology. Previous studies have documented the sublethal effects of environmental antibiotics on pollinators, but the vast majority focus on honeybees, with corresponding research on bumblebees remaining relatively scarce [15,16,17]. Meanwhile, existing studies mostly concentrate on changes in single physiological indicators, and the synergistic mechanisms of chloramphenicol disrupting bumblebee health through multiple pathways including neural gene expression, detoxification systems and gut microbiota remain poorly understood, limiting a holistic assessment of its ecological risks. To address these knowledge gaps, we hypothesized that sublethal chloramphenicol, despite causing no acute mortality, impairs bumblebee physiological health through three interconnected pathways: modulating the expression of neural, immune and detoxification-related genes; altering antioxidant and detoxifying enzyme activities; and disrupting gut microbiota homeostasis, thereby exerting latent chronic toxic effects. We conducted a 15-day chronic exposure experiment to test this hypothesis: we first monitored survival rate and food intake throughout the exposure period to verify the sublethal properties of the tested concentrations, and then detected molecular, biochemical and microbial indicators to explore the latent chronic toxic effects on bumblebees. We set two environmentally relevant dietary concentrations for exposure: 12 μg/L, representing low chloramphenicol residues in water and honey under conventional agricultural practices [18,19]; and 120 μg/L, corresponding to soil exposure levels that reflect realistic high-exposure scenarios in agricultural landscapes [20]. Both concentrations fall within the field-relevant dietary exposure range for pollinators, enabling an ecologically valid assessment of long-term chloramphenicol residue risks to bumblebees. This study aims to reveal the potential adverse effects and underlying mechanisms of sublethal chloramphenicol on the physiological health and behavioral adaptability of bumblebees, fill the knowledge gap in the response of bumblebees to chloramphenicol stress, provide a scientific basis for the comprehensive ecological risk assessment of agricultural antibiotic residues, and support the conservation of pollinator resources and the sustainable development of agricultural ecological environments.

## 2. Results

### 2.1. Effects of Chloramphenicol on Mortality and Food Intake of B. terrestris

**Figure 1:**
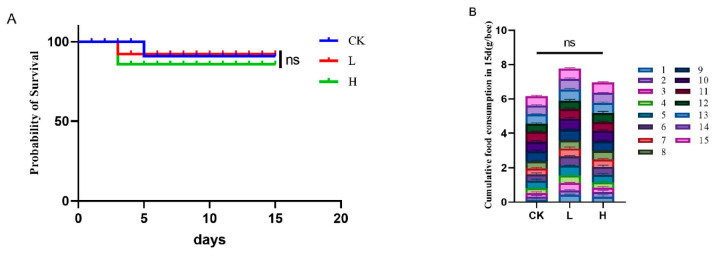
Survival rate and cumulative food intake of *B. terrestris* exposed to chloramphenicol for 15 days. (**A**) Survival curve changes of CK, L, and H groups during 15-day exposure. (**B**) Cumulative food intake of CK, L, and H groups over 15-day exposure. CK: control group (0 μg/L chloramphenicol); L: low-dose group (12 μg/L chloramphenicol); H: high-dose group (120 μg/L chloramphenicol). Results are expressed as mean ± SE; ns indicates no significant difference (*p* > 0.05).

Three treatment groups were set up in this study: control group (CK, 0 μg/L chloramphenicol), low-dose group (L, 12 μg/L chloramphenicol) and high-dose group (H, 120 μg/L chloramphenicol). The effects of 15-day chronic chloramphenicol exposure on the mortality and cumulative food intake of *B. terrestris* worker bees are presented in Figure 1A,B. After exposure to different sublethal chloramphenicol concentrations, the cumulative mortality of *B. terrestris* showed a slight upward trend with increasing chloramphenicol concentration, but no significant difference was detected among the CK, L, and H groups (*p* > 0.05). Similarly, there was no significant variation in cumulative food intake across all treatment groups (*p* > 0.05). These results indicate that within the concentration range set in this study, chloramphenicol does not exert acute toxic effects on the survival and feeding behavior of *B. terrestris* worker bees. These results confirm that the two chloramphenicol concentrations set in this study are indeed sublethal concentrations, which will not cause acute death or affect the foraging behavior of bumblebees, laying a prerequisite foundation for the subsequent exploration of sublethal effects on molecular, biochemical and microbial indicators. This result also reflects the concealment of antibiotic residue risks: environmentally relevant concentrations of chloramphenicol will not directly cause pollinator death, so their potential chronic toxicity is easily overlooked, which is the core scientific issue of this study.

### 2.2. Relative Expression of Memory-Related Genes in the Head of B. terrestris

**Figure 2:**
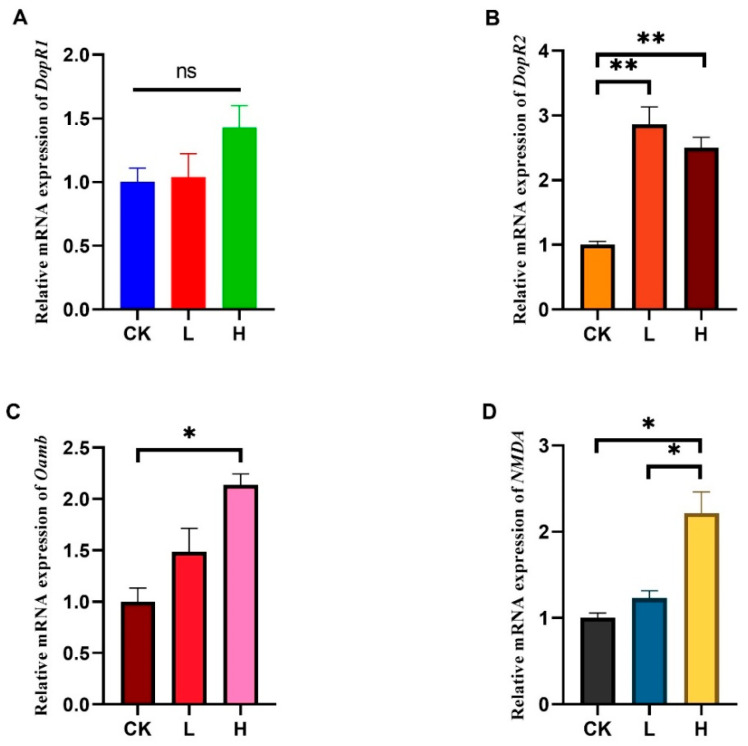
Effects of sublethal chloramphenicol exposure on the expression of learning and memory-related genes in *B. terrestris* heads. (**A**) Relative expression of *DopR1*. (**B**) Relative expression of *DopR2*. (**C**) Relative expression of *Oamb*. (**D**) Relative expression of *NMDA*. Results are expressed as mean ± SE. * *p* < 0.05, ** *p* < 0.01. ns, no significant difference.

*DopR1*, *DopR2*, *Oamb*, and *NMDA* genes are core regulatory genes closely associated with insect learning, memory, and neural signaling. Compared with the CK group, the relative expression level of *DopR1* in the L and H groups showed a slight upward trend with increasing chloramphenicol concentration, but the difference was not statistically significant (*p* > 0.05, Figure 2A), suggesting that *DopR1* expression is minimally affected by sublethal chloramphenicol exposure. The relative expression levels of *DopR2* were extremely significantly upregulated in both L and H groups compared with the CK group (*p* < 0.01, Figure 2B). *Oamb* expression was positively correlated with chloramphenicol concentration, with gradually increased expression levels in the L and H groups, and the H group exhibiting significantly higher than the CK group (*p* < 0.05, Figure 2C). *NMDA* expression was significantly higher in the H group than in the CK and L groups (*p* < 0.05, Figure 2D).

### 2.3. Effects of Chloramphenicol on the Expression of Immune and Detoxification Genes in the Abdomen of B. terrestris

**Figure 3:**
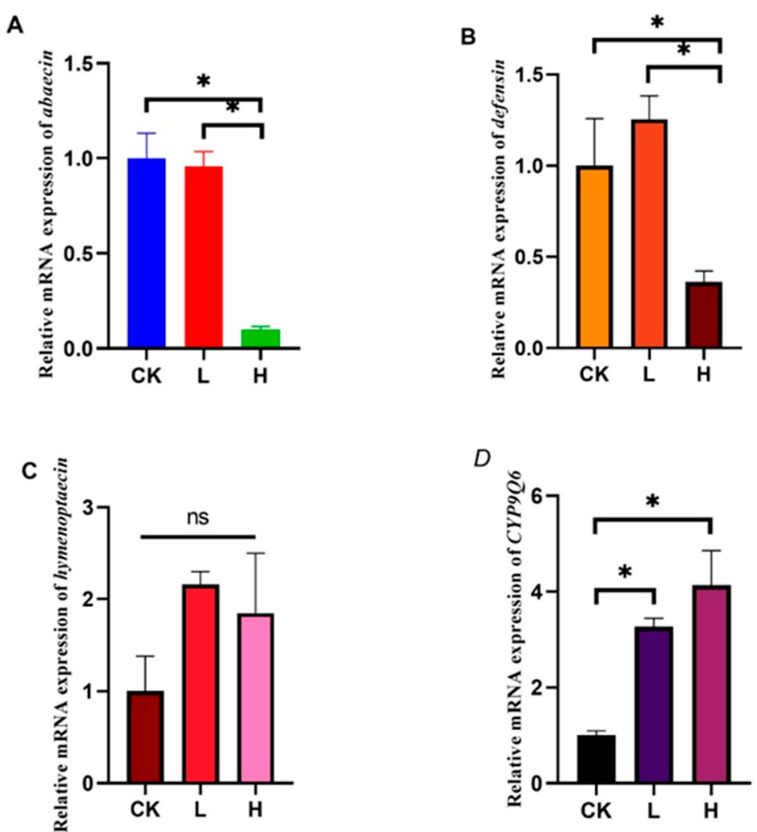
Effects of sublethal chloramphenicol exposure on the expression of immune and detoxification-related genes in *B. terrestris* abdomens. (**A**) Relative expression of *abaecin*. (**B**) Relative expression of *defensin*. (**C**) Relative expression of *hymenoptaecin*. (**D**) Relative expression of *cyp9Q6*. Results are expressed as mean ± SE. * *p* < 0.05; ns, no significant difference.

As illustrated in Figure 3A,B, high-dose chloramphenicol exposure significantly downregulated the expression levels of immune-related genes *abaecin* and *defensin* (*p* < 0.05), whereas no significant difference in *hymenoptaecin* expression was observed among all groups (*p* > 0.05, Figure 3C). For the detoxification-related gene cyp9Q6, both low and high concentrations of chloramphenicol significantly upregulated its expression level (*p* < 0.05, Figure 3D), indicating activation of the xenobiotic detoxification system in response to chloramphenicol stress.

### 2.4. Effects of Chloramphenicol Exposure on Enzyme Activities of B. terrestris

**Figure 4:**
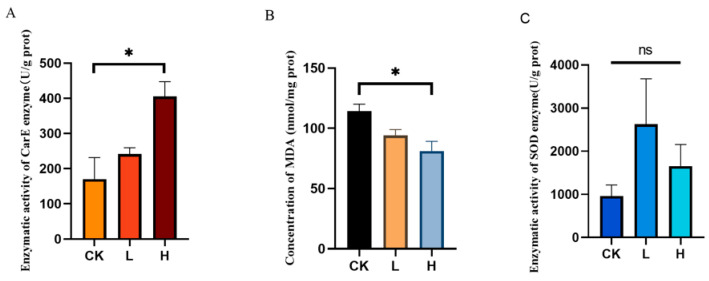
Effects of sublethal chloramphenicol exposure on detoxification and antioxidant enzyme indices in *B. terrestris*. (**A**) CarE activity. (**B**) MDA content. (**C**) SOD activity. Results are expressed as mean ± SE. * *p* < 0.05; ns, no significant difference.

After *B. terrestris* was exposed to high-dose chloramphenicol, the CarE activity was significantly increased (*p* < 0.05). Although the CarE activity in the L group showed an upward trend, it did not reach a significant difference level compared with the CK group (*p* > 0.05) (Figure 4A). The MDA content in the high-dose chloramphenicol treatment group was significantly lower than that in the control group (*p* < 0.05) (Figure 4B). There was no significant difference in SOD enzyme activity between the different chloramphenicol treatment groups and the control group (*p* > 0.05) (Figure 4C).

### 2.5. Effects of Chloramphenicol on the Structure and Function of Intestinal Microbial Community in Bumblebees

**Figure 5:**
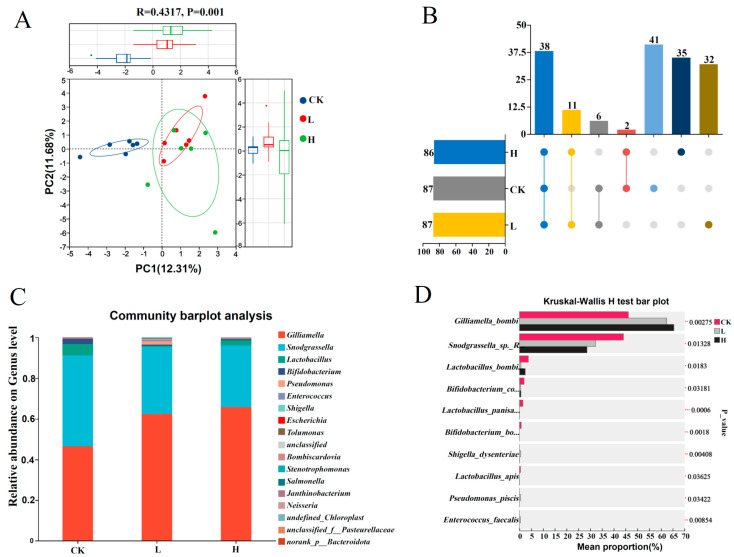
Effects of exposure to different concentrations of chloramphenicol on the intestinal microbes of *B. terrestris*. (**A**) PCA plots. (**B**) UPSET plot. (**C**) Bar chart of relative abundance at the genus level. (**D**) Kruskal–Wallis H test bar plot at the species level.

To analyze the overall effect of chloramphenicol exposure on the intestinal flora structure of *B. terrestris*, principal component analysis (PCA) was performed. The results showed that there was a significant separation of intestinal microbial community structure among different treatment groups (PCA: R = 0.4317, *p* = 0.001, Figure 5A). Alpha diversity analysis showed that there were no significant differences in Ace, Chao, Shannon, and Simpson indices among the groups (Figure S1). As shown in Figure 5B, there were 38 shared operational taxonomic units (OTUs) among the three groups, the control group (CK group) had 41 unique OTUs, the low-dose group (L group) had 32 unique OTUs, and the high-dose group (H group) had 35 unique OTUs. In addition, chloramphenicol treatment significantly changed the community composition of the intestinal microbes of *B. terrestris* at the genus level (Figure 5C). The results of community composition analysis at the species level showed (Figure 5D) that after chloramphenicol treatment, the abundance of *Gilliamella_bombi* was significantly increased (*p* = 0.00275), while the abundances of *Snodgrassella_sp*, *Lactobacillus_bombicola*, *Bifidobacterium_commune*, and *Lactobacillus_panisapium* were significantly decreased (*p* = 0.01328, *p* = 0.0183, *p* = 0.03181, *p* = 0.0006, respectively), which confirmed that chloramphenicol had a selective effect on specific intestinal genera of *B. terrestris*.

## 3. Discussion

Chloramphenicol, a broad-spectrum antibiotic, is widely used in animal husbandry and agricultural production. Its residues can enter pollinators via pollen, nectar and other dietary routes, exerting potential adverse effects on their survival, physiological functions and intestinal microecological homeostasis. As a widely commercialized bumblebee species, *B. terrestris* is extensively used for crop pollination in facility agriculture in China and plays an important role in agricultural production. In the present study, a chronic toxicological exposure assay was conducted to systematically investigate the effects of chloramphenicol on the mortality, food consumption, memory-immune gene expression, antioxidant enzyme activity and intestinal microbial community structure of *B. terrestris*. The overall results of this study support our research hypothesis: sublethal chloramphenicol can interfere with the physiological state of bumblebees at multiple levels without affecting survival and foraging, thus posing potential health risks. Based on the experimental findings and previous relevant studies, the toxicological effects and underlying mechanisms of chloramphenicol exposure on this pollinator were thoroughly discussed, which provides a reliable scientific basis for assessing the ecological risks of chloramphenicol residues to pollinating insects.

The results of the chronic exposure experiment in this study showed that after 15 days of exposure to different concentrations of chloramphenicol, there were no significant differences in the mortality and food intake of *B. terrestris*, indicating that within the concentration range set in this experiment, chloramphenicol would not have obvious acute toxic effects on the survival and feeding behavior of *B. terrestris* in the short term. We detected the survival rate throughout the exposure period mainly for two purposes: first, to verify that the two tested concentrations are truly sublethal doses, ensuring that the subsequent physiological and molecular changes are sublethal effects rather than secondary reactions caused by acute death; and second, to confirm that environmentally relevant concentrations of chloramphenicol will not directly cause the death of bumblebees, so their potential chronic toxic risks are highly hidden and easy to be ignored, which highlights the necessity of this study. This is consistent with the results of some studies, which found that low-concentration antibiotic exposure had no significant effect on the mortality of honeybees. This may be because pollinators have a certain tolerance to low-dose antibiotics, and chloramphenicol mainly acts on the ribosome synthesis process of bacteria, with weak direct toxicity to insect’s own cells [21]. However, it should be noted that this experiment only set a 15-day exposure period. Whether long-term low-dose chloramphenicol exposure will affect the long-term survival indicators of *B. terrestris* such as lifespan and reproductive capacity through cumulative effects still needs further long-term follow-up experiments to verify.

The differential expression of these neuromodulatory genes reflects the potential molecular response of the bumblebee nervous system under chloramphenicol stress, which may further affect neural function and behavioral performance, but the actual behavioral effects need to be verified by targeted behavioral assays in subsequent studies. The possible reason for the upregulation of *DopR2* is the stress protection of nerve cells, which attempts to maintain motor and cognitive functions by enhancing dopamine signals, but long-term high expression will lead to excessive activation of the dopamine pathway [22]. *DopR2* is a core receptor for learning and memory and synaptic plasticity, and its increased expression level may be an attempt by nerve cells to compensate for damage by enhancing synaptic plasticity, but long-term excessive activation will lead to neuroexcitotoxicity [23]. *DopR2* is a core neurotransmitter receptor that mediates stress responses, locomotor activity, and foraging behavior in insects. Short-term overexpression can compensatively maintain motor ability, stress response and foraging activity, but long-term excessive activation will cause bumblebee restlessness, behavioral instability and excessive energy consumption [24]. Under chloramphenicol stress, the co-upregulation of *DopR2*, *Oamb* and *NMDA* gene expression in bumblebees reflects that octopamine, dopamine and glutamate systems are all involved in neural stress response and toxic compensation; this overall neurotransmitter system disorder will lead to abnormal motor excitement, impaired learning and memory function, increased risk of neuroexcitotoxicity, and ultimately reduce the individual adaptability and colony stability of bumblebees.

The immune and detoxification system of insects is an important barrier to resist the invasion of external harmful substances. Among them, the *abaecin*, *defensin* and *hymenoptaecin* genes are key immune-related genes in insects [25], which are involved in the synthesis of antimicrobial peptides, the activation of immune cells and the transmission of immune signals, respectively. The *cyp9Q6* gene belongs to the cytochrome P450 family and is mainly responsible for the metabolic detoxification of exogenous substances in the body [26]. This study found that high-dose chloramphenicol exposure significantly reduced the expression levels of the *abaecin* and *defensin* genes, had no significant effect on the expression of the *hymenoptaecin* gene, and significantly upregulated the expression of the *cyp9Q6* gene. This result indicates that chloramphenicol exposure can inhibit some immune functions of *B. terrestris* and activate its detoxification system to cope with drug stress. The changes in the expression of antimicrobial peptide genes and detoxification genes indicate the molecular response of bumblebees to chloramphenicol stress, suggesting that the immune and detoxification pathways may be affected. The actual changes in immune capacity and detoxification efficiency need to be further confirmed by physiological functional tests. The downregulation of *abaecin* and *defensin* gene expression may lead to a decrease in the synthesis of antimicrobial peptides in *B. terrestris*, reduce its resistance to pathogens such as bacteria and fungi, and increase the risk of infection. The significant upregulation of the *cyp9Q6* gene indicates that *B. terrestris* reduces the accumulation of chloramphenicol in the body by enhancing the metabolic capacity of exogenous substances, thereby reducing the toxic effect of the drug, which is also an adaptive defense mechanism of *B. terrestris* against chloramphenicol exposure. However, the expression of the *hymenoptaecin* gene had no significant change, which may be because this gene is mainly involved in specific immune responses, and chloramphenicol has a weak regulatory effect on it. The specific mechanism still needs further in-depth study.

CarE enzyme, SOD enzyme activity and MDA content are important indicators reflecting xenobiotic detoxification capacity and oxidative status in insects. Among them, CarE is a core phase I detoxification enzyme responsible for hydrolytic metabolism of xenobiotics, the SOD enzyme is a key enzyme for scavenging oxygen free radicals, and MDA is the end product of lipid peroxidation. Its content change reflects the degree of cell damage [27,28,29]. The experimental results showed that high-dose chloramphenicol exposure significantly increased CarE activity, significantly decreased MDA content, but had no significant effect on SOD enzyme activity. This indicates that chloramphenicol exposure can induce an oxidative stress response in *B. terrestris*. Under high-dose chloramphenicol treatment, the elevation of CarE activity may be an adaptive detoxification response of *B. terrestris* to antibiotic stress. By enhancing the metabolic clearance of chloramphenicol and its intermediates, it reduces oxygen free radical accumulation and lipid peroxidation level, thereby alleviating cell damage. The lack of a significant change in SOD enzyme activity may be because the regulation of the SOD enzyme by chloramphenicol is concentration-dependent, or its scavenging of oxygen free radicals mainly depends on other antioxidant enzymes. In addition, the significant decrease in MDA content also indicates that under high-dose chloramphenicol treatment, the antioxidant system of *B. terrestris* is activated, effectively reducing lipid peroxidation damage. However, CarE activity did not increase significantly under low-dose chloramphenicol exposure, likely because the low dosage was insufficient to induce a notable compensatory upregulation of the xenobiotic detoxification system.

The stability of the intestinal microbial community is closely related to insect nutrient absorption and immune defense [30]. As a broad-spectrum antibiotic, chloramphenicol residues have a selective impact on intestinal microorganisms. PCA analysis in this study showed that there was a significant separation of intestinal microbial community structure between different chloramphenicol treatment groups and the control group (PCA: R = 0.4317, *p* = 0.001), but there were no significant differences in alpha diversity analysis (Ace, Chao, Shannon and Simpson indices) (see Figure S1). OTU analysis showed that there were 38 core OTUs in the three groups, and the number of independent OTUs in the control group was more than that in the low- and high-dose groups, suggesting that chloramphenicol mainly changes the flora composition rather than species richness. Species-level analysis showed that chloramphenicol treatment significantly increased the abundance of *Gilliamella_bombi* (*p* = 0.00275) and significantly decreased the abundance of beneficial bacteria such as *Snodgrassella_sp*. and *Lactobacillus_bombicola* (*p* < 0.05). This will break the intestinal microecological balance, weaken the host’s immune and nutrient metabolism functions, and may also have a synergistic effect with the abnormal physiological indicators mentioned earlier, providing a microecological basis for evaluating the ecological risks of chloramphenicol to pollinators.

The selective effect of chloramphenicol on intestinal flora is mainly achieved by inhibiting the ribosome synthesis of sensitive bacteria (such as *Lactobacillus* and *Bifidobacterium*) [31], which provides a survival advantage for drug-resistant bacteria (such as *Gilliamella_bombi*) and leads to an imbalance in the symbiotic competition relationship between flora. The stable existence of core OTUs indicates that there is a core flora tolerant to chloramphenicol in the intestine of *B. terrestris*, which can maintain the basic physiological functions of the intestine, while the reduction in unique OTUs reflects the extinction of sensitive and specific flora, which may indirectly affect the specific physiological functions of the host. Although this change in flora structure did not affect species diversity, the imbalance between the decrease in the abundance of beneficial bacteria and the proliferation of drug-resistant bacteria may further aggravate the physiological damage of *B. terrestris* in the long term and also provide an important entry point for subsequent research on the association between intestinal flora and host physiological functions, as well as the analysis of the toxicological mechanism of chloramphenicol.

## 4. Materials and Methods

### 4.1. B. terrestris

In May 2025, 10 early-stage colonies of *B. terrestris* were purchased from Biote Biotechnology Co., Ltd (Kunming, China). All colonies were reared in a dark rearing room maintained at 29 ± 1 °C with 55% relative humidity. After newly emerged worker bees appeared in the colonies, those within 24 h of eclosion were selected and placed in customized plastic cages. The worker bees were acclimated with 50% sucrose solution fed via 5 mL syringes for 1 day, and the sucrose solution was replaced daily.

### 4.2. Reagent Preparation

Chloramphenicol (product No.: ST1150-5g, molecular weight 323.13, purity ≥ 98%) was purchased from Beyotime Biotechnology Co., Ltd (Shanghai, China). A total of 2.4 mg of chloramphenicol powder was accurately weighed using an analytical balance and dissolved in 1 L of 50% sucrose solution to prepare a stock solution with a chloramphenicol concentration of 2400 μg/L. Subsequently, gradient dilutions were performed to obtain the high-dose group (H group, 120 μg/L) and low-dose group (L group, 12 μg/L), while 50% sucrose solution without chloramphenicol was used as the control group (CK group, 0 μg/L). All prepared solutions were stored in a refrigerator at 4 °C until use.

### 4.3. Chronic Toxicity Experiment

The newly emerged worker bees were randomly divided into three experimental groups. Each experimental unit (micro-colony) consisted of 5 worker bees, serving as one biological replicate, with six biological replicates established per group. Each group was fed ad libitum with sucrose solution containing the corresponding concentration of chloramphenicol (CK: 0 μg/L, L: 12 μg/L, H: 120 μg/L). The weight of the sucrose solution in each syringe was weighed daily to calculate the cumulative food intake of each micro-colony. Simultaneously, the number of dead *B. terrestris* worker bees was recorded daily to determine the cumulative mortality rate under each treatment concentration.

### 4.4. Sample Collection and Treatment

After 15 days of rearing, 3 surviving worker bees were randomly selected from each rearing cage (each biological replicate), and all subsequent statistical analyses took the rearing cage as the statistical unit. Individual bees from the same cage were not regarded as independent biological replicates, which effectively avoided the problem of pseudo-replication. The collected bees were anesthetized by cooling on ice. Immediately after anesthesia, the bees were transferred to a sterile dissecting tray, and sterile dissection was performed strictly on an ultra-clean workbench: The intestinal tissue was separated first and quickly frozen in liquid nitrogen for subsequent intestinal microbial community analysis. Then, the head and abdomen of the worker bees were separated respectively, placed into sterile 1.5 mL centrifuge tubes, and labeled properly. The head and abdomen tissues in the centrifuge tubes were cut into pieces separately, and 500 μL of Milli-Q ultra-pure water and 2 pieces of 3 mm magnetic beads were added to each tube, followed by thorough grinding and mixing in a tissue grinder. After grinding, the head tissue homogenate was used for total RNA extraction to detect the expression of associative memory-related genes. The abdominal tissue homogenate was evenly divided into 2 parts: one part was used for total RNA extraction, and the other part was used for the determination of detoxification and antioxidant-related enzyme activities.

### 4.5. Gene Expression Detection

Total RNA was extracted from head and abdomen samples using the Trizol method, with the specific steps as follows: take the ground head and abdomen tissues, add 500 μL of RNA extraction solution (catalog No.: G3013-100ML, purchased from Sevier Biological Service Co., Ltd., Wuhan, China) to each sample, mix thoroughly with a vortex oscillator, and let stand at room temperature for 5 min; then add 100 μL of chloroform (operation in the dark) and let stand for 3 min; centrifuge at 12,000 rpm at 4 °C for 15 min, absorb the supernatant, and add 250 μL of isopropanol and mix well, then let stand for 10 min; centrifuge at 12,000 rpm at 4 °C for 10 min and discard the supernatant; and add 500 μL of 75% ethanol, centrifuge at 12,000 rpm at 4 °C for 2 min, discard the ethanol, and after the ethanol is completely volatilized, add 50 μL of ultra-pure enzyme-free water to dissolve the RNA. After extraction, the concentration and purity of total RNA were detected by a microspectrophotometer (NanoDrop 2000, Thermo Fisher Scientific, Waltham, MA, USA). The A260/A280 ratio of all RNA samples ranged from 1.8 to 2.0, and the RNA concentration ranged from 210 ng/μL to 830 ng/μL, with good integrity and purity, which met the requirements of subsequent reverse transcription and qPCR experiments. Then, the concentration and integrity of RNA were detected, and the RNA was stored at −80 °C for later use. The Hifair^®^ III 1st Strand cDNA Synthesis SuperMix for qPCR kit (catalog No.: 11141ES60, purchased from Yeasen Biotechnology Co., Ltd., Shanghai, China) was used to reverse transcribe total RNA into cDNA. Relevant reagents were added according to the kit instructions, and reverse transcription was performed in a PCR instrument with the following reaction conditions: 25 °C for 5 min, 55 °C for 25 min, 85 °C for 5 min, and incubation at 4 °C. The reverse-transcribed cDNA was stored at −20 °C for later use. Real-time fluorescence quantitative PCR (qPCR) was performed using cDNA as the template, with the reaction conditions set as follows: pre-denaturation at 94 °C for 30 s; then enter the cycle stage: denaturation at 94 °C for 5 s, annealing at 60 °C for 34 s, totaling 40 cycles. The qPCR data were analyzed for relative expression using the 2^−∆∆Ct^ method, and β-actin was selected as the internal reference gene to correct experimental errors. The primer sequences of each target gene and internal reference gene are shown in Table S1.

### 4.6. Enzyme Activity Determination

The activities of carboxylesterase (CarE), superoxide dismutase (SOD) and the content of malondialdehyde (MDA) were determined using corresponding commercial kits, strictly following the kit instructions. The specific steps are as follows: take the separated abdomen of *B. terrestris*, add tissue extraction solution, and prepare homogenate with a tissue grinder; centrifuge the homogenate at 8000 rpm at 4 °C for 10 min, absorb the supernatant as crude enzyme solution, and place it on ice for later use. The BCA Protein Quantification Kit (catalog No.: G2006-200T, purchased from Sevier Biological Service Co., Ltd., Wuhan, China) was used to determine the sample protein concentration, with a detection wavelength of 562 nm. Before subsequent enzyme activity assays, the protein concentration of all samples was adjusted to 0.5 mg/mL to ensure consistent loading. The detection kits and corresponding detection wavelengths for each indicator are as follows: CarE assay kit (catalog No. BC0845, Solarbio Science & Technology Co., Ltd., Beijing, China), detection wavelength 450 nm; SOD assay kit (catalog No. BC5156, Solarbio Science & Technology Co., Ltd., Beijing, China), detection wavelength 450 nm; and MDA assay kit (catalog No. BC6415, Solarbio Science & Technology Co., Ltd., Beijing, China), detection wavelength 532 nm. During the determination, add the sample supernatant and each reaction reagent in turn according to the kit requirements, mix thoroughly, incubate at the specified temperature until the reaction is completed, and use a microplate reader (Multiskan™ FC, Thermo Fisher Scientific, Waltham, USA) to detect the absorbance value of each reaction system at the corresponding characteristic wavelength. Each sample was set with 6 technical replicates, and the blank control group was used for zero adjustment. According to the standard curve and the measured absorbance value, the CarE and SOD activities and MDA content of each group were calculated according to the kit instructions.

### 4.7. Intestinal Flora Analysis

The hindgut samples of surviving *B. terrestris* were selected for intestinal microbial community analysis, and the experimental technology was supported by Majorbio Biomedical Technology Co., Ltd. (Shanghai, China). All dissection procedures were performed on a super-clean bench to avoid environmental contamination. All surgical instruments (forceps, scissors) were autoclaved at 121 °C for 20 min before use, and a new set of sterile instruments was replaced for each sample to avoid cross-contamination. 16S rRNA gene PCR amplification and library construction were performed according to the Majorbio standard protocol. The amplification primers were 27F (5′-AGRGTTYGATYMTGGCTCAG-3′) and 1492R (5′-RGYTACCTTGTTACGACTT-3′), which were used to amplify the full length of the 16S rRNA gene. After PCR reaction, quantification, quality control and purification, sequences with similarity ≥ 97% were clustered into operational taxonomic units (OTUs). UPARSE 7.1 software was used to cluster sequences into OTUs according to 97% similarity and remove chimeras. An RDP classifier (https://sourceforge.net/projects/rdp-classifier/, accessed on 3 July 2026) was used to compare the Silva 16S rRNA gene database (v138) for OTU taxonomic annotation, with a confidence threshold of 70% for subsequent bacterial α-diversity analysis. The Unweighted Pair-Group Method with Arithmetic Means (UPGMA) clustering and PCA were used to evaluate β-diversity and the bacterial species diversity, and their relative abundance between the control group and each treatment group were compared.

### 4.8. Data Analysis

All experimental data were expressed as mean ± standard error (SE). The K-S test was used to verify the normality of the data in each group, and the results showed that all data conformed to the normal distribution. The Brown–Forsythe test was used to test the homogeneity of variances, and *p* > 0.05 indicated that the data had homogeneity. One-way analysis of variance (ANOVA) combined with Tukey’s test (SPSS 26.0 software) was used for inter-group comparison; when *p* < 0.05, non-parametric test was used. The log-rank (Mantel–Cox) test was used to compare the differences in survival curves between groups (GraphPad Prism 9.5 software).

## 5. Conclusions

The chronic exposure experiment in this study showed that within the experimental concentration range, chloramphenicol did not have a significant impact on the short-term mortality and food intake of *B. terrestris*, which confirmed the sublethal characteristics of the tested concentrations. Consistent with our research hypothesis, sublethal chloramphenicol could interfere with the physiological state of *B. terrestris* at multiple levels: it regulated the expression of genes related to learning and memory, and immunity and detoxification, induced changes in antioxidant enzyme activities, and selectively altered the intestinal microbial community structure by targeting the abundance of specific bacterial genera without altering alpha diversity. These changes at the molecular, biochemical and microecological levels suggest potential health risks to bumblebees, but the actual functional impacts on behavior, immunity and colony fitness need to be further verified by more targeted physiological and behavioral experiments. This study provides a multi-dimensional scientific basis for evaluating the ecological risks of chloramphenicol residues to pollinators, identifies the limitations of short-term exposure and single-concentration experiments, and puts forward prospects for subsequent research on long-term and combined exposure.

## Data Availability

The original contributions presented in this study are included in the article and Supplementary Materials. Further inquiries can be directed to the corresponding authors.

## Supplementary Materials

The following supporting information can be downloaded at: https://www.mdpi.com/article/10.3390/ijms27136004/s1.
