# Supplementary material for: Sublethal Concentration of Chloramphenicol Threatens the Health of Bombus terrestris by Regulating Gene Expression, Altering Enzyme Activity and Disrupting Gut Microbiota
Source: Int J Mol Sci. 2026 Jul 4;27(13):6004. doi: 10.3390/ijms27136004 (PMC13360809; doi:10.3390/ijms27136004)
Supplement: Supplementary file 1 [file ijms-27-06004-s001.zip › Table S1.pdf]

Table S1 The sequences of primers

Gene	Primer sequence （5′-3′）
DopR1	F:CCCGTAATGTATGATGATGGTAAAG
	R:CGATGCAGGGCACGTAAAA
DopR2	F:GGAGGAAGTGCCAGAGGACA
	R:TCACGAACAGGGGTAAGTAGAAAC
NMDA	F:GAGTCTTCATAGTGGTCGGTGTTG
	R:TGTCTCGCCAGTTCCATCTTT
Oamb	F:AGGGCGATGAAAATGGGG
	R:AAGAAAGGAAGCCAGCAGAGG
abaecen	F:CCACGACCGGGACAATCTA
	R:CCAGGGTTTGGTAATGGGTATG
defensin	F:TGCCGATAGACAAAGAAGAGTGA
	R:TTTGCCCATGCTGAGACAGT
CYP9Q6	F:GATTCACCACGATCCCAAGC
	R:CCGAGACCAAACGGCATAA
hymen	F:GCTGCCAGAATTGAACCTGA
	R:CCGCTCAATGGTTTCTTTCC
β-actin	F: CGACTACCTCATGAAGATT
	R: CGACGTAACAAAGTTTCTC
