# Supplementary figures and images for: Sublethal Concentration of Chloramphenicol Threatens the Health of Bombus terrestris by Regulating Gene Expression, Altering Enzyme Activity and Disrupting Gut Microbiota
Source: Int J Mol Sci. 2026 Jul 4;27(13):6004. doi: 10.3390/ijms27136004 (PMC13360809; doi:10.3390/ijms27136004)

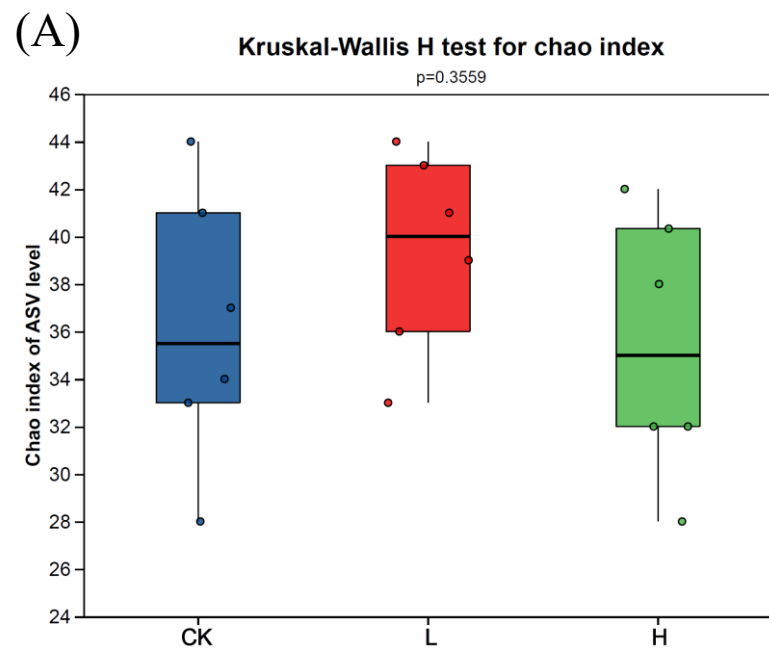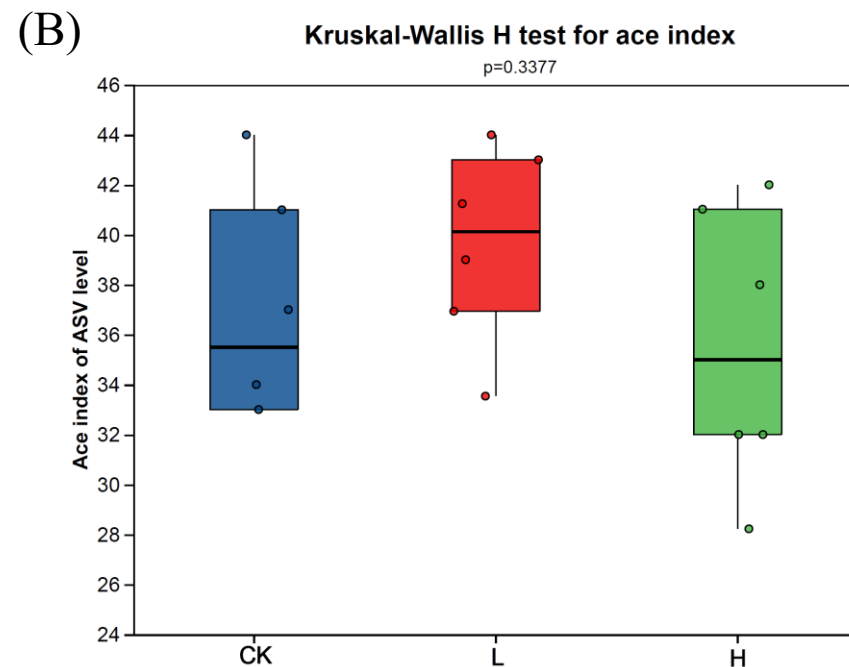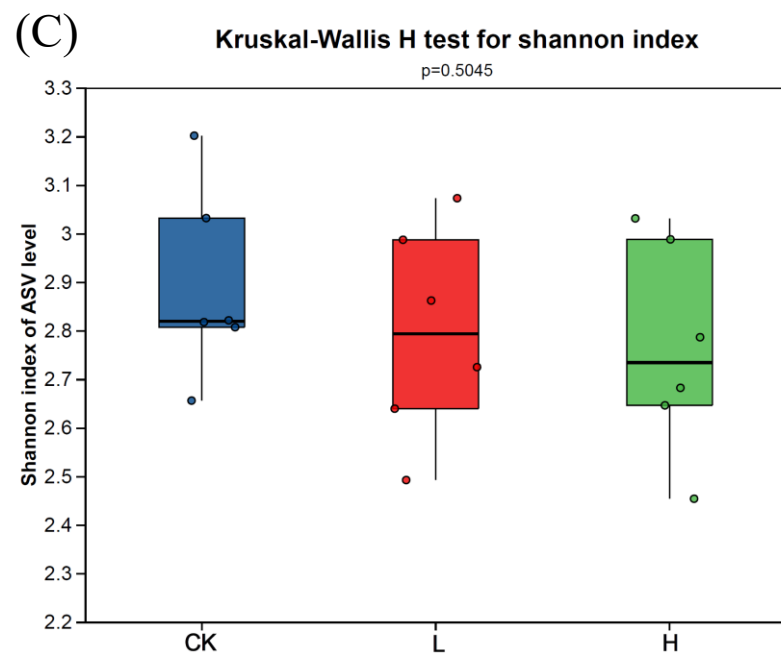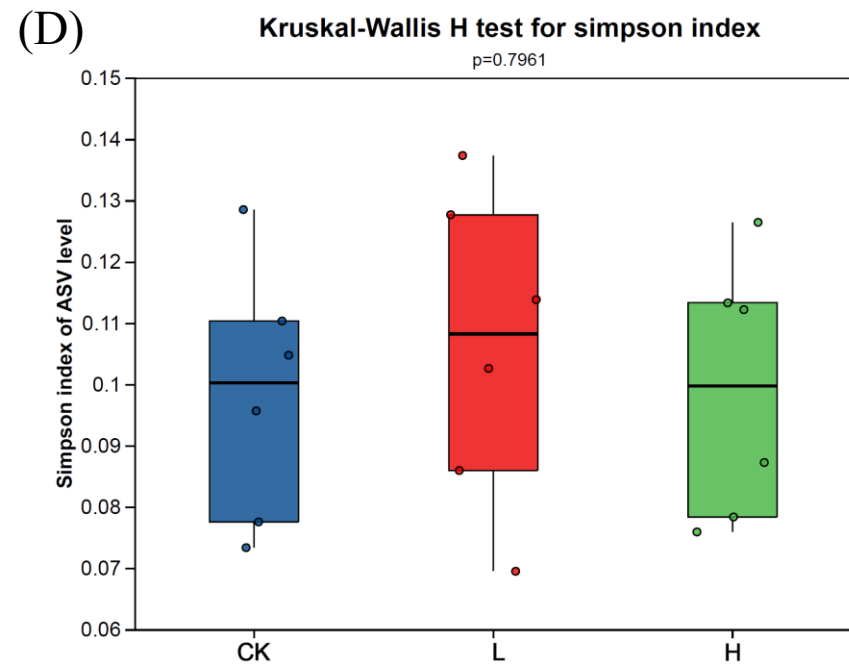

Supplement: Supplementary file 1 [file ijms-27-06004-s001.zip › Figure S1.pdf]
